# The effect of natriuretic peptides and bradykinin on development of brain oedema after ischemic stroke

**DOI:** 10.1186/2050-6511-16-S1-A88

**Published:** 2015-09-02

**Authors:** Marina Dobrivojević, Katarina Špiranec, Dunja Gorup, Igor Erjavec, Srećko Gajović, Aleksandra Sinđić

**Affiliations:** 1 Department of Histology and Embryology, School of Medicine, University of Zagreb, Zagreb, 10 000, Croatia; 2 Croatian Institute for Brain Research, School of Medicine, University of Zagreb, Zagreb, 10 000, Croatia; 3 Department of Anatomy, Histology and Embryology, Faculty of Veterinary Medicine, University of Zagreb, Zagreb, 10 000, Croatia; 4 Department of Anatomy, School of Medicine, University of Zagreb, Zagreb, 10 000, Croatia; 5 Department of Physiology and Immunology, School of Medicine, University of Zagreb, Zagreb, 10000, Croatia

## Background

Ischemic stroke is characterized by a rapid loss of brain function due to disturbance in blood supply to a part of the brain. Due to fixed intracranial space, any increase in intracranial fluid volume, or progressive brain oedema formation, contributes to further deterioration of the already impaired brain function. Bradykinin (BK), which levels increase during ischemic stroke, promotes blood–brain barrier permeability and raises intracranial capillary blood pressure, leading to brain oedema formation. Furthermore, BK induces glutamate release from neurons and astrocytes via activation of BK receptor type 2. suggesting involvement of BK in glutamate neurotoxicity. It has been recently shown that humans without functional natriuretic peptides (NPs) suffer from massive stokes [1,2].

NPs can reduce brain oedema and have a neuroprotective role in acute ischemic stroke as well as during recovery after stroke. Although mechanisms are still not clear, it appears that NPs enhance angiogenesis, neurogenesis and oligodenrogenesis [3,4]. One of the possible beneficiary effects of NPs during the stroke could be an inhibition of BK pathological function.

## Materials and methods

Aim of our study is to determine beneficial effects of the NPs in stroke development in murine model (middle cerebral artery occlusion – MCAO). The symptoms of the stroke are determined by behavioural studies. The sizes of the lesion and brain oedema are established by μCT. Furthermore, we determined the effects of NPs on the BK signalling pathway in primary culture of neurons and astrocytes using whole cell patch clamp experiments to measure membrane potential and measurements of intracellular Ca^2+^ concentration.

## Results

In primary isolated astrocytes and neurons, BK binding to type 2 receptor, leads to an increase in intracellular C^2+^ concentration of astrocytes and neurons, followed by activation of Ca^2+^-dependent Cl^-^ channel which depolarized the cell membrane. Agonists of guanylate cyclase A, partially guanylate cyclase C but not guanylate cyclase B inhibited the effects of BK at the membrane potential and intracellular Ca^2+^ concentration via regulators of G protein signalling. *In vivo* experiments showed that urodilatin inhibited development of stroke symptoms, the formation of the ischemic lesion and brain oedema.

## Conclusion

The results of this research show the existence of a natural antagonist of the BK receptor type 2 in the mouse brain, and the possible use of NPs in treatment of the stroke.
